# Ten simple rules for teaching an introduction to R

**DOI:** 10.1371/journal.pcbi.1012018

**Published:** 2024-05-16

**Authors:** Ava M. Hoffman, Carrie Wright

**Affiliations:** 1 Biostatistics Program, Fred Hutchinson Cancer Center, Seattle, Washington, United States of America; 2 Department of Biostatistics, Johns Hopkins University, Baltimore, Maryland, United States of America; Dassault Systemes BIOVIA, UNITED STATES

## Introduction

The demand for data science skills across disciplines has led to massive growth of data-related programs in universities, standalone courses, and other learning materials [1]. While demand is high, program administrators often struggle to find instructors [2]. Many learners end up teaching themselves and climbing a very steep learning curve [3]. On the other hand, scientists with deep research backgrounds often end up teaching with little experience or guidance. We believe better resources for busy and/or less experienced instructors are part of the solution to meeting the demand for programming skills education.

The Open Educational Resources (OER) movement has propelled data science forward by improving the quality, depth, and availability of free materials [4]. This includes textbooks, tutorials, vignettes, datasets, software tools, case studies, and of course, programming courses. Despite ongoing challenges, such as awareness and access to computers, democratizing educational resources makes high-quality content available to a global audience. This in turn nurtures collaboration and innovation. As data science continues to influence all aspects of our lives, we must consider key takeaways for adopting materials across classrooms and contexts.

Here, we provide guidance to instructors tasked with teaching beginner level programming, specifically an introduction to the R language. Our perspective comes from co-teaching over 300 professionals, graduate students, and undergraduate interns at institutions like Johns Hopkins School of Public Health, University of Washington, and Fred Hutchinson Cancer Center. Our learners usually lack a computer science background and often juggle full-time employment and coursework. The 10 rules we present distill our key reflections from our experiences teaching R. We hope these rules will help you plan your course structure, incorporate general guidance and approaches, and understand specific pitfalls learners are likely to encounter.

## Rule 1: Make it intensive

Most of us feel the strain of day-to-day distractions like responding to and reading emails, Slack messages, and various other pings. These forms of communication, while (probably) important, can be distracting and get in the way of deep work [5]. Programming is about problem solving, which can be frustrating or even impossible if you are thinking about the next task on your to-do list. Instead of learning R in small chunks, we suggest a condensed format for introducing R where learners’ distractions are minimized. Intensive courses can lead to better learner experiences and outcomes, such as greater course effectiveness [6], lower perceived stress [7], improved focus [7,8], and greater student success rates [8]. As the instructor, you should also fully commit to being present for learners by minimizing meetings and unrelated correspondence where possible during a condensed course.

**Fig 1:**
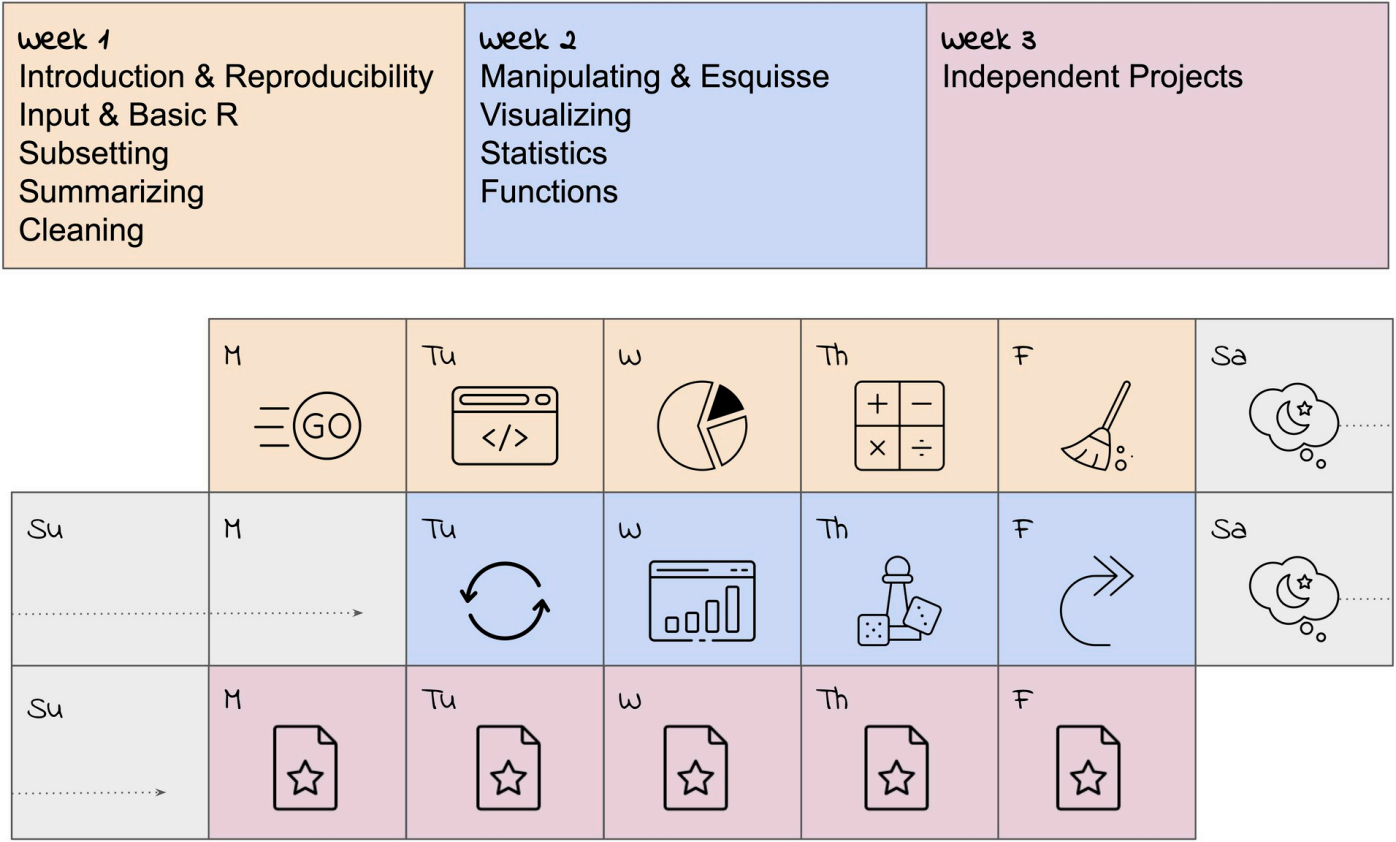
Our “Introduction to R” course at Johns Hopkins University takes place over 9 days, followed by time for independent projects. In week 1, an introduction is given followed by topics in reproducibility, data input, basic R, data subsetting, summarizing, and cleaning. Week 2 consists of data manipulating, an introduction to a visualization GUI (for example, Esquisse—[13]), programmatic visualization, statistics, and functions. Week 3 allows learners to complete projects. Note that week 2 typically includes a free Monday to observe Martin Luther King, Jr. Day or Juneteenth.

Learning a programming language is not unlike learning a spoken language. It requires focus, commitment, and practice [3]; some degree of immersion can also be helpful [9–11]. For example, our course “Introduction to R for Public Health Researchers” (Fig 1) [12] takes place during the Johns Hopkins University Summer and Winter Institutes, over 9 days of 3 hour sessions each. Sessions are recorded to help participants review the material and to maximize accessibility. After the 9 interactive days, learners have a week in which to submit assignments and a final project (see Rule 6). Based on our experience and student feedback, we believe our format effectively balances immersion in R with measures that largely prevent learners from feeling overwhelmed.

## Rule 2: Teach as a team

No one instructor has expertise in every aspect of R programming. Consider teaching as a team, where multiple instructors take turns diving into topics. A teaching team provides a broader range of skills, knowledge, and personalities when going over materials. We all explain solutions to problems a bit differently, which can help “unlock” programming principles for a wider array of learners. Team teaching also has other benefits, such as greater professional and social support for instructors [14].

No matter how down-to-Earth your team is, many learners find programming intimidating [15]. Learners often have many questions or do not know what questions to ask. Leverage tools like Slack or Zoom chat to boost communication and peer collaboration. These platforms enable learners to ask questions, seek clarifications, and learn from peers’ conversation threads in a way that can feel more familiar [16]. Asynchronous communication on platforms like Slack can complement the live sessions and accommodate different learning styles. Ensure there are ample opportunities for questions, both directly to your team, and in public forums with peers.

Working as a team of instructors makes it easier to manage these communication channels. For example, while one instructor leads the lecture and demonstrations, another can provide real time assistance via Zoom chat or Slack. This approach creates a supportive learning atmosphere and ensures that learners have access to timely guidance throughout their learning journey. This kind of support is critical for an inclusive and diverse learner cohort [1]. It also ensures the instructor leading the demonstrations is able to stay on-topic and stick within anticipated time constraints.

## Rule 3: Teach reproducibly using dynamic documents

It can be challenging to maintain a current version of R, let alone keep up with the constant updates in the R ecosystem. Thoughtful planning with a version control system, such as Git and GitHub, makes updating easier and minimizes lead time ahead of the class. To ensure consistency of materials across many publishing platforms, we designed an in-house GitHub-based tool called OTTR (ottrproject.org) [17] to automate rendering materials, including our website [12], from plain text R Markdown documents. We encourage you to use GitHub’s other features, such as issues and projects, to track changes and ideas in real-time. This simplifies the process of identifying and resolving sticking points for learners in the course materials as they emerge, and before you forget.

Similarly, we recommend using R Markdown or Quarto file formats during live demos and labs. Using these tools encourages beginners to follow best practices, including organizing code into sections and annotating code, by default. It might be much faster for you, a seasoned coder, to quickly enter something in the console, but beginners often lack the intuition for what code should be “saved” and what code is disposable. Learners should practice saving all their code in reproducible documents where they can also take notes and record their thoughts throughout the process.

## Rule 4: Prioritize intuition over memorization

Programming is much more than just memorizing functions. During an introductory programming course, learners should start to develop an intuition for the language. In other words, they will begin to understand the grammar of how functions, objects, and chunks of code are ordered and oriented. However, learners will often get bogged down in details and feel overwhelmed. It is important to reiterate to learners that a key goal is to practice and gain familiarity and intuition for R programming.

We strongly recommend equipping learners with this expectation from the beginning. Being clear about this goal can help shift their attitude and anticipations about their experience to be more positive and growth-oriented. They are also more likely to grasp crucial skills for their future R programming journey, like interpreting documentation and seeking assistance. In the age of code-ready large language models like ChatGPT, it is more essential to teach how to interpret, test, and debug, rather than recall specific functions.

To encourage intuition, we include summaries of key functions at break points, such as before breaking out into a coding lab or at the end of a module. This helps learners focus on application of key functions and behaviors rather than recall. Summary pages and cheatsheets that match the course content ([18], see “Cheatsheets” section) can also serve as key reference points throughout the course.

Another strategy is having your class embrace the practical solution of “Googling it.” Teach learners how to search appropriately, reference documentation, and assess code from crowd-sourced forums like StackOverflow with a critical eye [19]. You can also explain that professionals check documentation regularly, as packages are updated frequently. We believe sharing how you search for answers is part of being an authentic teacher, which can lead to more meaningful and deeper student learning [20].

## Rule 5: Boost live code and lab time

Live coding with an instructor makes it easier for beginners to understand the programming process, code debugging, and good coding practices [21–23]. Although it might be challenging to implement for large class sizes, hands-on experience is vital for learners to integrate new information, make mistakes, and learn from one another. We suggest prioritizing live coding demonstrations and allocating ample lab time for learners to practice [24]. In student feedback, this is consistently highlighted as our strongest course component.

During live demonstrations, check your ego at the door and embrace errors made in front of the class. This can be a fantastic opportunity to ask the class, “who can spot where I’ve made a mistake?”, “let’s guess what is causing this issue”, or even “let’s consult the documentation for clues.” For team-taught courses, it can be useful to divide the class into smaller groups for lab sessions. We have found that learners have different preferences; some want more guidance during lab, others want to try everything themselves first, while others need more one-on-one attention. Instructors can take on different levels of guidance to match these preferences.

## Rule 6: End the course with a project

Like learning to drive a car, real-world practice is arguably the best way to become proficient. R programming should be a practical, goal-oriented skill, with responsible practices in mind [25]. To reinforce this, you should center the course around a comprehensive project [26]. Embracing a project-oriented approach enables learners to apply concepts in real-world scenarios, including their own (nonsensitive) data, fostering deeper comprehension, curiosity, and motivation [27]. This works particularly well for graduate students and professionals who are more likely to have their own data available. You can further encourage creativity and autonomy in project selection to cater to individual interests and goals. Undergraduate students or learners who lack data will benefit from more guidance [28], such as repositories where they can find publicly available data (e.g., as appear in [29]). Ultimately, projects should test application of knowledge through a defined rubric, which when refined ahead of time, makes grading less stressful.

Ensure learners have easy access to the many online resources where they can customize their projects and continue their development (e.g., [18]). Where possible, take the time to meet with members of the class individually and support them in discovering the right next steps. Approach these conversations with an open mind; discussions might include applying for jobs in both academia and industry, what to do with their specific type of data, or how to translate commands from other platforms (such as SAS or Stata) or interfaces (such as Shiny). Remember that you now serve as a data science role model for your learners.

## Rule 7: Get (and give) feedback often

Classroom surveys can be an excellent way to understand learners’ backgrounds, build rapport, and make the course more inclusive, especially in an online setting [30–32]. Use a free platform, such as Google Forms, to collect information about learners’ interests. For example, you might ask about their majors/areas of study, why they are taking the class, and their level of previous experience with computers more generally. This information helps ground the course, helping you determine how much time to spend on fundamentals versus more advanced or niche topics. Understanding learners’ general interests can help you choose more relevant data sets and examples.

Ensure learners are able to provide feedback throughout the duration of the course. We use a “pulse check” feedback form which can be taken multiple times throughout the course [33]. Learners are encouraged to provide feedback daily, though this is not required. We also make the form anonymous by default, with the option to fill in an email address or name if the learner wishes. Feedback forms can reveal how learners are feeling generally (such as on a Likert 1–10 scale), but can also help you understand what went well, what was challenging, how the pace feels, or what they would like to see covered or reviewed in more detail (such as via free text). For example, we received feedback early on in our course that learners were more interested in cleaning character data (i.e., with stringr) and handling missing data (i.e., with naniar). In future iterations, we dove into these topics in more detail instead of other topics, like “date” data types. Anonymous forms also provide the opportunity for learners to alert you to more serious issues, such as problems related to the code of conduct.

When providing feedback, strive to cultivate an inclusive and supportive environment to encourage continuous growth and improvement. Instead of simply correcting errors, encourage practice, resourcefulness, and problem-solving skills, as they are essential in the real-world application of R programming. This includes showing learners how they might find the correct answers in documentation or vignettes, search StackOverflow.com, or use AI tools like ChatGPT or Phind to translate a chunk of code. Importantly, if learners locate code elsewhere, they should be able to interpret, at a very general level, what is happening and why. As with any teaching, be very clear about the criteria by which learners will be graded, if applicable (see Rule 6).

## Rule 8: Commit to tidyverse or base R

In R, you can help new learners build a strong foundation by committing to either the Tidyverse ecosystem or base R from the outset. Switching between related languages or language “dialects” can erode learner confidence and cause confusion [21,34]. For example, learners often struggle with when to extract a column of a dataset as a vector using dplyr::pull(). Mixing in base $ notation further muddies the waters. A new programmer can accomplish their end goal using either readr::read_csv (Tidyverse) or read.csv() (base R) and does not need to know both when starting out.

The choice of R ecosystem depends both on your experience and comfort level, as well as the goals of your student audience. For example, our audience typically consists of researchers and practitioners in the public health sphere who have no prior R experience. These learners are more interested in data cleaning, analysis, and visualization. The Tidyverse works well for these goals and is valued by instructors for its readability, consistency, and user community [34]. However, if your learners are more interested in software development, dependency minimization, optimization, or mathematical applications, base R might be more appropriate. Some researchers have found base R easier for learners [35], while others found no difference [36].

## Rule 9: Start with data wrangling

An introduction to R programming should focus on data wrangling and basic statistics rather than diving into complexities of data types, classes, and functional programming. Most R users are interested in analyses and data transformation; very few users describe themselves as software developers [37]. The reality is that most R learners will not be writing software, at least not right away. Data wrangling, or the process of cleaning, preparing, transforming, and/or manipulating data, is typically the most time consuming part of data analysis and is a prerequisite for visualization, reporting, and other statistical insights [38].

Data wrangling is a practical and essential skill in most fields and allows learners to work with interesting, real-world datasets from the start. Once learners gain an intuition for R through data cleaning and manipulation techniques, they will be better prepared to explore more advanced topics. As Rule 8 suggests, how long the instructor spends on data wrangling does depend somewhat on your audience. Learners who will take their R skills back to applied fields will benefit from spending more time with data wrangling. Learners with experience in other programming languages might be more eager to move on to advanced topics more quickly. Get to know your class with surveys (Rule 7) so you can adjust accordingly.

## Rule 10: Know the common pitfalls

Explicitly pointing out common mistakes can help learners avoid them without added anxiety. It can also help them focus on actually learning the intended material. We encourage you to include resources specifically addressing common mistakes, introduce common mistakes when starting a new topic, and show common mistakes during demonstrations. Encouraging learners to share and describe mistakes often helps other learners feel more comfortable and confident. These moments also provide an opportunity to discuss why these mistakes happen. Having taught hundreds of learners, we see many pitfalls repeatedly. We have summarized these here and on our course Help page (Table 1) [39].

**Table 1:** Common pitfalls encountered while teaching introductory R content, with suggested solutions and/or mitigation.

Pitfall	Solution/Mitigation
Learners often make typos or forget to load packages, resulting in Error in x : could not find function "y".	Encourage them to check their spelling and library() statements frequently.
Learners miss closing parentheses, quotation marks, and backticks.	Emphasize how IDEs like RStudio can help minimize these errors, but they will still need to check for these. There is no “auto-correct” feature. Show them to esc if a “+” appears at the console command line.
R is not an “in place” language. Learners often miss reassigning objects to update them.	Remind them that they have to “save” an object in memory by assigning it, e.g.: data <- data + 1 instead of just printing data + 1 to the console.
Knowing when to use "quotation marks" and `backticks` can be tricky even for experienced R users.	Make sure learners are used to testing code with and without these. Providing a resource about the different uses, especially in Tidyverse functions, can help [40].
Base R and the Tidyverse functions don’t always mix and match well, for example, count(iris$species) will not work.	Avoid teaching learners about both ecosystems simultaneously to reduce confusion. Indicate where they might encounter challenges with combining the two (see Rule 8).
Learners are often confused by functions that require a vector or a column vs. those that are more flexible and can use a vector or a data frame.	Providing a guide can help [41].
R is generally very forgiving about spacing; however, pipes and ggplot2 plus signs cannot start a new line.	Pointing out this nuance during demonstrations is extremely helpful to learners. RStudio’s “Reformat Code” feature can be helpful for spacing.

## Conclusions

Over 5 course sessions and additional workshops, we have co-taught R to hundreds of learners. We have consistently earned overall course and overall instructor ratings in the outstanding *range (over 3.6 on a 4-point scale with 70% or greater response rate) for our courses*). Using student feedback, we have earned these scores through continuous improvement and collaborative refinement. We feel that these rules strike the right balance in making R approachable for learners with limited computational experience, while also cultivating skills for rigorous and ethical scientific practices.

Although our experience has inspired us to follow these “rules,” we still face a number of challenges. The learning curve for R is steep, and many learners wish for a gentler on-ramp. However, aside from prescribing self-guided pre-work and offering assurance, we have yet to find a consistently effective way to address the root of this challenge without sacrificing content. Interestingly, learners who dislike the learning curve generally feel the class was very rewarding and that they learned a lot. Ultimately, you will encounter many trade-offs when instructing, and you should consider benefits and drawbacks thoughtfully with respect to your audience.

We encourage you to explore additional resources and references to further enhance your teaching methods. These include not only academic journals, but also teaching materials and lesson plans on platforms like the Data Carpentry and RStudio Education, as well as blogs, data science conferences, and package vignettes. Together, we can contribute to a thriving community and collection of resources, ensuring that learners have access to high-quality programming education.

Much of the advice we give to brand new R programmers applies to teaching R as well. Make mistakes and try new things. Google things and read documentation. Explore new communication approaches, such as via social media. Leverage the community and reach out to colleagues for support. Build off of open-source resources to grow your teaching plan. Tell the story of your R journey, and enjoy passing what you know along to the next wave of R users.
